# An Eye Destroyed by a Bird-Shot

**Published:** 1852-09

**Authors:** 


*An Eye Destroyed by a Bird-Shot.*—Mr. —— was hunting birds on the 11th March, 1843, when, in order to secure their game, he took one side of the field and his brother the other, the distance between them being supposed sufficiently great not to incur any risk in firing towards each other. His brother fired, and a single shot seems to have reached Mr. ——. This passed through the cornea and lodged within the globe of the eye. There being but little pain, and several days passing without much inflammation, the patient flattered himself that the accident would not prove very serious. The pain, however, began to be acute, inflammation rapidly increased, the eye swelled out enormously, and the patient was finally relieved by excision of the cornea, which allowed the disorganized humors to escape with the shot in their midst. Recovery took place in the usual time, and a glass was substituted, which very effectually obviates the deformity.

The reporter finds in the Southern Medical and Surgical

Journal, for 1838, (vol. 2, p. 647,) several cases recorded, in which Prof. Dugas resorted to excision of the cornea for the purpose of relieving great local pain and constitutional disturbance consequent upon a disorganization of the contents of the eye. Whenever the eye is irretrievably lost and proves a source of serious annoyance, Prof. D. thinks that it should be at once emptied, both as a measure of relief and as a security against sympathetic disease in the sound organ. He has never found any bad effects from such a course.—*Southern Med. & Surg. Jour.*

